# Vegan Diet, Greenhouse Gas Emissions, and Cumulative Energy Demand: A Secondary Analysis of a Randomized Clinical Trial

**DOI:** 10.1001/jamanetworkopen.2025.43871

**Published:** 2025-11-17

**Authors:** Hana Kahleova, Arathi Jayaraman, Brighid McKay, Laura Chiavaroli, Songhee Back, Ilana Fischer, Reagan Smith, Richard Holubkov, Neal D. Barnard

**Affiliations:** 1 Physicians Committee for Responsible Medicine, Washington, DC; 2 Department of Nutritional Sciences, Temerty Faculty of Medicine, University of Toronto, Toronto, Ontario, Canada; 3 Toronto 3D Knowledge Synthesis and Clinical Trials Unit, Clinical Nutrition and Risk Factor Modification Centre, St Michael’s Hospital, Toronto, Ontario, Canada; 4 Li Ka Shing Knowledge Institute, St Michael’s Hospital, Toronto, Ontario, Canada; 5 School of Medicine, University of Utah, Salt Lake City; 6 George Washington University School of Medicine and Health Sciences, Washington, DC

## Abstract

This secondary analysis of a randomized clinical trial examined the environmental effect of adopting a low-fat vegan diet on greenhouse gas emissions and cumulative energy demand.

## Introduction

Dietary changes can reduce the environmental footprint,^1^ in addition to improving health measures.^2^ This secondary analysis of a randomized clinical trial assessed the environmental effect of adopting a low-fat vegan diet, hypothesizing that greenhouse gas emissions (GHGE) and cumulative energy demand (CED), which estimates total energy used to produce, process, package, transport, store, and dispose of the waste of food, would be reduced.

## Methods

This study was conducted between January 2017 and February 2019 in Washington, DC, and the study methods have been previously described (trial protocol in Supplement 1).^2^ This ad hoc secondary analysis is reported in accordance with the CONSORT guideline. The protocol was approved by the Chesapeake Institutional Review Board. All participants provided written informed consent.

Participants were randomly assigned to a vegan or control group in a 1:1 ratio (eFigure in Supplement 2). The vegan group was asked to follow an ad libitum low-fat vegan diet consisting of fruits, vegetables, grains, and legumes, while the control group was requested to make no diet changes. At baseline and week 16, a 3-day dietary record was completed by each participant and analyzed by a registered dietitian certified in the Nutrition Data System for Research.^3^

Intakes from dietary records were linked to the US Department of Agriculture Food Commodity Intake Database^4^ and the database of Food Impacts on the Environment for Linking to Diets^5^ by 3 independent reviewers (A.J., B.M., and S.B.) who were blinded to group assignment. Linking accuracy was verified by a senior researcher (L.C.), who was also blinded to group assignment. A repeated measure analysis of variance was used by a statistician blinded to dietary interventions. All results are presented as means with 95% CIs. SAS version 9.4 (SAS Institute) was used to conduct analyses, and statistical significance was set at *P* < .05. Data were analyzed from June to July 2025.

## Results

Of 3115 people screened by telephone, 244 overweight adults (211 women [86.5%] and 33 men [13.5%], mean [SD] age, 54.8 [11.7] years) met participation criteria and were randomly assigned to the vegan (122 [50.0%]) or control (122 [50.0%]) group. The analysis included 223 individuals who completed the study (117 vegan [52.5%] and 106 control [47.5%] group).

The vegan group had an associated decrease in GHGE by 1313 (95% CI, −1486.6 to −1139.4) g carbon dioxide equivalent GHGE (CO_2_-eq) per person-day (*P* < .001), compared with a reduction by 314 (95% CI, −596.0 to −32.0) g CO_2_-eq per person-day in the control group (effect size, −999 [95% CI −1329 to −669] g CO_2_-eq/person-day; *P* < .001) (Figure and Table). This associated decrease in the vegan group was mainly attributable to reduced meat consumption, followed by reduced dairy consumption. The vegan group had an associated decrease in CED by 8193.8 (95% CI, −9369.3 to −7018.4) kJ per person-day (*P* < .001), compared with no change in the control group (effect size, −6767 [95% CI −8718 to −4817] kJ/person-day; *P* < .001) (Figure and Table). Again, most of the reduction was attributable to reduced meat intake, followed by reduced egg consumption.

**Figure.**
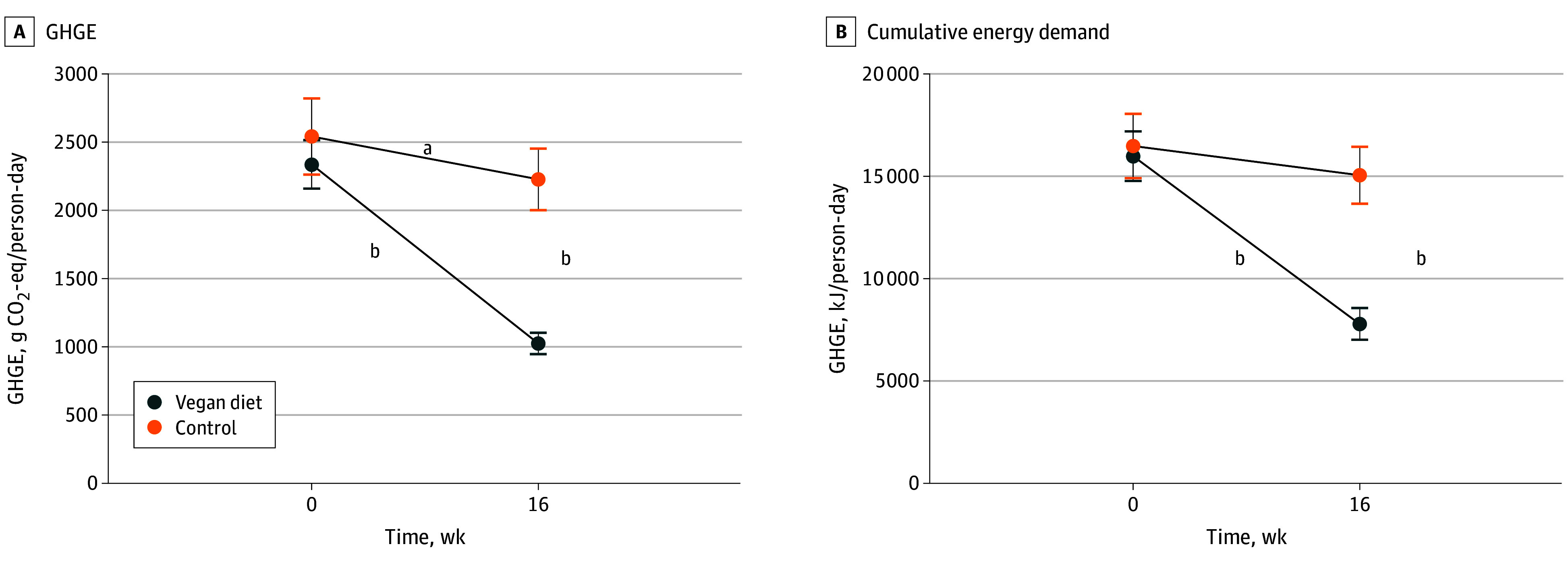
Changes in Total Greenhouse Gas Emissions (GHGE) and Cumulative Energy Demand in the Vegan and Control Groups From Baseline to Week 16 The data are shown as means with 95% CIs. CO_2_ indicates carbon dioxide. ^a^*P* < .05. ^b^*P* < .001.

**Table.** Changes in Greenhouse Gas Emissions (GHGE) and Cumulative Energy Demand (CED) From Specific Food Groups at Baseline and Week 16 in the Control and Vegan Groups, Ranked According to the Magnitude of Their Impact on Changes in the Metrics

Food groups	Group, mean daily intakes (95% CI)						Effect size	*P* value
	Control			Vegan				
	Week 0	Week 16	Change	Week 0	Week 16	Change		
**GHGE, g CO_2_-eq/person-day**								
Total	2540.5 (2262.3 to 2818.6)	2226.5 (2000.9 to 2452.0)	−314.0 (−596.0 to −32.0)^a^	2337.0 (2159.5 to 2514.4)	1023.9 (945.9 to 1102.0)	−1313.0 (−1486.6 to −1139.4)^b^	−999.0 (−1328.7 to −669.4)	<.001
Meat	939.5 (758.9 to 1120.1)	864.7 (695.4 to 1034.0)	−74.8 (−263.9 to 114.2)	803.7 (662.2 to 945.1)	4.0 (0 to 7.9)	−799.7 (−941.3 to −658.1)^b^	−724.9 (−959.9 to −489.9)	<.001
Dairy products	486.3 (346.4 to 626.2)	404.6 (325.5 to 483.7)	−81.7 (−224.2 to 60.7)	454.1 (372.4 to 535.7)	6.0 (1.7 to 10.2)	−448.1 (−530.3 to −365.9)^b^	−366.4 (−530.2 to −202.6)	<.001
Eggs	106.4 (79.9 to 132.8)	100.8 (75.5 to 126.1)	−5.5 (−32.9 to 21.8)	107.4 (84.1 to 130.8)	0.5 (−0.1 to 1.0)	−107.0 (−130.3 to −83.7)^b^	−101.4 (−136.9 to −65.9)	<.001
Added fats	65.6 (51.7 to 79.5)	69.4 (52.6 to 86.2)	3.8 (−12.8 to 20.5)	61.3 (50.4 to 72.1)	17.1 (12.9 to 21.2)	−44.2 (−55.7 to −32.7)^b^	−48.0 (−68.2 to −27.9)	<.001
Nuts and seeds	33.9 (24.8 to 42.9)	28.5 (22.0 to 34.9)	−5.4 (−15.6 to 4.7)	29.1 (20.8 to 37.4)	8.8 (6.4 to 11.2)	−20.3 (−28.9 to −11.7)^b^	−14.9 (−28.0 to −1.7)	.03
Grains	183.9 (161.7 to 206.1)	122.0 (106.7 to 137.3)	−61.9 (−85.6 to −38.2)^b^	207.4 (184.3 to 230.5)	177.5 (160.1 to 195.0)	−29.9 (−57.7 to −2)^a^	32.1 (−4.3 to 68.4)	.08
Fruit	99.4 (79.0 to 119.8)	96.3 (77.0 to 115.6)	−3.1 (−27.9 to 21.7)	101.2 (82.5 to 119.8)	139 (122.0 to 156.1)	37.8 (16.5 to 59.2)^b^	40.9 (8.6 to 73.3)	.01
Legumes	27.2 (19.7 to 34.7)	23.2 (16.5 to 29.9)	−4.0 (−11.6 to 3.6)	25.8 (19.2 to 32.5)	71.8 (57.7 to 86.0)	46.0 (31.6 to 60.4)^b^	50.0 (33.8 to 66.2)	<.001
Vegetables	164.8 (146.3 to 183.3)	158.0 (141.4 to 174.6)	−6.8 (−29.2 to 15.6)	161.8 (144.5 to 179.2)	228.3 (204.4 to 252.3)	66.5 (40.2 to 92.8)^b^	73.3 (39.0 to 107.7)	<.001
**CED, kJ/person-day**								
Total	16 473.6 (14 901.9 to 18 045.3)	15 047.0 (13 656.7 to 16 437.3)	−1426.6 (−2995.7 to 142.5)	15 976.1 (14 765.6 to 17 186.7)	7782.3 (7010.3 to 8554.3)	−8193.8 (−9369.3 to −7018.4)^b^	−6767.2 (−8717.5 to −4816.9)	<.001
Meat	5511.3 (4531.7 to 6490.8)	5388.5 (4390.3 to 6386.6)	−122.8 (−1256 to 1010.4)^b^	5370.1 (4550.4 to 6189.8)	84.0 (−23.6 to 191.7)	−5286.1 (−6115.6 to −4456.5)^b^	−5163.3 (−6560.5 to −3766.1)	<.001
Eggs	640.3 (481.0 to 799.6)	607.0 (454.8 to 759.2)	−33.3 (−198.2 to 131.5)^b^	646.8 (506.2 to 787.4)	2.9 (−0.3 to 6.1)	−644.0 (−784.2 to −503.7)^b^	−610.6 (−824.5 to −396.8)	<.001
Dairy products	2260.8 (1861.4 to 2660.2)	2087.5 (1701.8 to 2473.2)	−173.3 (−615.7 to 269.1)^b^	2346.9 (1966.3 to 2727.4)	32.4 (9.0 to 55.8)	−2314.5 (−2698.1 to −1930.9)^b^	−366.4 (−530.2 to −202.6)	<.001
Added fats	366.8 (293.8 to 439.8)	384.5 (299.5 to 469.6)	17.7 (−68.3 to 103.7)^b^	336.1 (280.1 to 392.1)	101.3 (75.5 to 127.1)	−234.8 (−295.7 to −173.9)^b^	−252.5 (−357.4 to −147.6)	<.001
Nuts and seeds	193.5 (143.1 to 243.9)	165.5 (125.1 to 205.9)	−28.0 (−83.5 to 27.6)^b^	164.4 (115.3 to 213.4)	44.3 (31.2 to 57.3)	−120.1 (−170.8 to −69.5)^b^	−92.2 (−166.8 to −17.5)	.02
Grains	1130.2 (993.1 to 1267.4)	740.3 (649.8 to 830.8)	−390.0 (−535.1 to −244.8)^c^	1276.2 (1132.5 to 1419.8)	1045.7 (945.3 to 1146.1)	−230.5 (−400.7 to −60.3)^c^	159.5 (−63.0 to 381.9)	.16
Legumes	136.7 (99.1 to 174.3)	116.7 (83.1 to 150.2)	−20.1 (−58.2 to 18.1)^b^	129.8 (96.4 to 163.2)	361.0 (289.8 to 432.1)	231.2 (159.0 to 303.4)^b^	251.2 (169.9 to 332.6)	<.001
Fruit	805.3 (648.7 to 962)	779.6 (629.2 to 930.1)	−25.7 (−211.8 to 160.4)^b^	804.6 (668.4 to 940.8)	1082.2 (952.6 to 1211.8)	277.6 (120.3 to 434.9)^b^	303.3 (62.7 to 544.0)	.01
Vegetables	1159.8 (1028.4 to 1291.2)	1100.5 (985.5 to 1215.4)	−59.3 (−218.4 to 99.7)^b^	1133.9 (1013.3 to 1254.5)	1598.8 (1432.1 to 1765.4)	464.9 (285.0 to 644.8)^b^	524.2 (285.5 to 763.0)	<.001

Abbreviation: CO_2_-eq, carbon dioxide equivalent.

^a^ *P* < .05.

^b^ *P* < .001.

^c^ *P* < .01.

## Discussion

The EAT-Lancet Commission demonstrated that animal foods, particularly red meat, have an outsized impact on GHGE and energy use, compared with grains, legumes, fruits and vegetables.^6^ A systematic review of 14 different dietary patterns showed that a vegan diet was associated with the largest reduction of GHGE.^1^

The strengths of the current study include a randomized, parallel design, which accounted for seasonal impacts. The study also has limitations. Food consumption was based on self-reported diet records. The participants were research volunteers and may not represent the general population. In this randomized study, adopting a low-fat vegan diet was associated with a significant reduction in GHGE and CED, important drivers of climate change.

## Notes

Supplement 1.
Trial Protocol

Supplement 2. **eFigure. **Participant Flow Chart

Supplement 3.
Data Sharing Statement
