# Supplementary material for: Vegan Diet, Greenhouse Gas Emissions, and Cumulative Energy Demand: A Secondary Analysis of a Randomized Clinical Trial
Source: JAMA Netw Open. 2025 Nov 17;8(11):e2543871. doi: 10.1001/jamanetworkopen.2025.43871 (PMC12625681; doi:10.1001/jamanetworkopen.2025.43871)
Supplement: Supplement 2. — eFigure. Participant Flow Chart [file jamanetwopen-e2543871-s002.pdf]

Supplemental Online Content

Kahleova H, Jayaraman A, McKay B, et al. Vegan diet impact on greenhouse gas emissions and cumulative energy demand. *JAMA Netw Open*. 2025;8(11):e2543871. doi:10.1001/jamanetworkopen.2025.43871

eFigure. Participant Flow Chart

This supplemental material has been provided by the authors to give readers additional information about their work.

© 2025 Kahleova H et al. *JAMA Network Open*.

eFigure. Participant Flow Chart

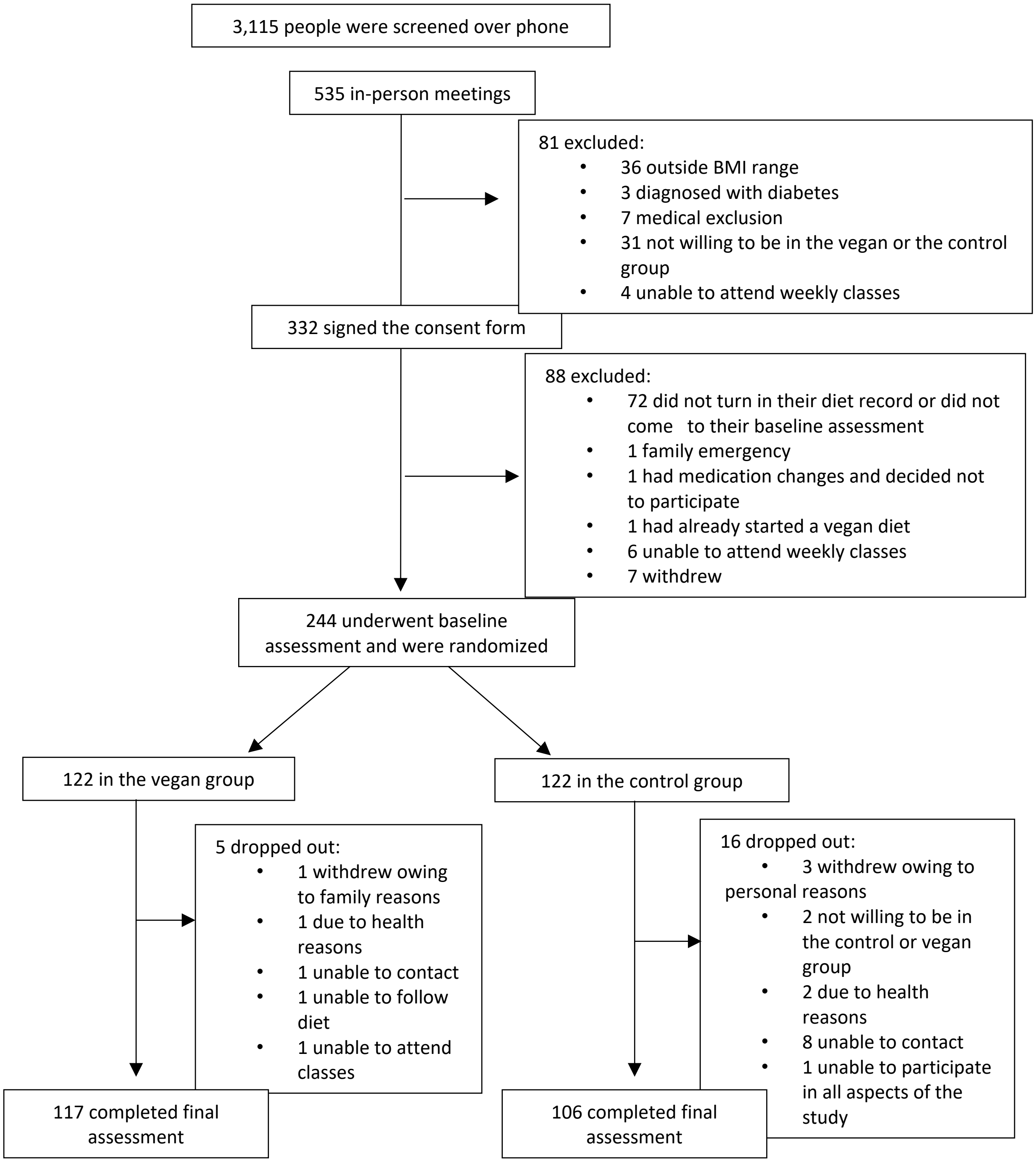

© 2025 Kahleova H et al. *JAMA Network Open*.
