# Supplementary material for: Vegan Diet, Greenhouse Gas Emissions, and Cumulative Energy Demand: A Secondary Analysis of a Randomized Clinical Trial
Source: JAMA Netw Open. 2025 Nov 17;8(11):e2543871. doi: 10.1001/jamanetworkopen.2025.43871 (PMC12625681; doi:10.1001/jamanetworkopen.2025.43871)
Supplement: Supplement 3. — Data Sharing Statement [file jamanetwopen-e2543871-s003.pdf]

Data Sharing Statement

Data
Additional Information: NCT02939638
Data available: Yes
Data types: Deidentified participant data
How to access data: Data will be made available upon request at hana.kahleova@gmail.com.
When available: With publication

Supporting Documents
Document types: None

Additional Information
Who can access the data: Researchers whose proposed use of the data has been approved.
Types of analyses: For reviews and meta-analyses.
Mechanisms of data availability: After approval of a proposal.
